# Is the policy of “no active re-warming in the emergency department” adequate for therapeutic hypothermia after cardiac arrest

**DOI:** 10.1186/1757-7241-21-S1-S22

**Published:** 2013-05-28

**Authors:** JW Keep, Q Adelasoye, JR Pallett, S Calvert

**Affiliations:** 1 Kings College Hospital, London, UK

## Objectives

The National Institute for Health and Clinical Excellence (NICE) recommend that after out of hospital cardiac arrest (OHCA) in patients with return of spontaneous circulation (ROSC), therapeutic hypothermia is induced “as soon as possible” to maintain core body temperature at 32-34°C for 12-24 hours.[1] Surface or internal cooling techniques are technically challenging in the Emergency Department (ED), instead we have a policy of ‘No active re-warming’ in our department. This study aimed to assess the adequacy of this policy.

## Methods

A retrospective observational cohort study of OHCA patients achieving ROSC in the ED was performed. Patients < 18 years and those admitted for palliative care were excluded. Transit times from arrival in the ED to Intensive Care Unit (ICU) either directly or via percutaneous coronary intervention (PCI) were recorded. In addition, body temperature in the ED and on arrival in ICU with the time of active cooling was obtained.

## Results

Between Jan 2009 and June 2012, 258 patients with OHCA were identified. Notes were unavailable for 3 cases. 14 were excluded as < 18 years. Of the remaining 241, 71 achieved ROSC (29%) and 167 died in the ED. Of the survivors: 62 were admitted to ICU, (44 directly from the ED and 18 via PCI) and 9 admitted for palliative care on the ward. 1 case of hyperpyrexia was excluded. Time from arrival at the ED to direct admission in ICU was 2hr 58mins (n=26). This time increased to a mean of 3hr 50mins when patients went via PCI (n=9). The mean temperature on arrival in the ED for all patients was 35.15°C. Mean temperature on arrival in ICU after direct transfer from the ED was 35.24°C and on arrival in ICU after PCI was 35.30°C. 13 patients had therapeutic hypothermia in ICU of which 69% survived to discharge. Mean time to initiation of cooling from arrival in the ED was 6hr 6mins (1hr 53mins – 12hr 27mins).

## Conclusions

Our policy did not achieve hypothermia by the time of ICU admission. Transit to PCI after ROSC increases the mean time to arrival in ITU by approximately 1 hour and does not appear to affect ICU arrival temperature. Cooling techniques are required in the ED phase of the patient pathway to initiate therapeutic hypothermia as soon as possible after ROSC.
